# Editorial: Nature-based social prescriptions for improving health and wellbeing

**DOI:** 10.3389/fpsyg.2022.1095064

**Published:** 2022-12-12

**Authors:** Carly Wood, Lauriane Suyin Chalmin-Pui, Nina Smyth, Jakub Rajcani

**Affiliations:** ^1^School of Sport, Rehabilitation and Exercise Sciences, University of Essex, Colchester, United Kingdom; ^2^Science Team, Royal Horticultural Society, Wisley, United Kingdom; ^3^Department of Landscape Architecture, The University of Sheffield, Sheffield, United Kingdom; ^4^Department of Psychology, University of Westminster, London, United Kingdom; ^5^Department of Psychology, Comenius University, Bratislava, Slovakia

**Keywords:** green social prescribing, health, wellbeing, nature-based activities, ill-health

## Introduction

Social Prescribing offers a means by which third sector organizations can provide non-medical sources of support within local communities to address mental, psychosocial, and socio-economic needs through provision of a range of “social” activities (Chatterjee et al., 2018). Nature-based or green social prescriptions are one type of social prescription which aim to improve health and wellbeing through exposure to, and direct interaction with natural environments (Robinson and Breed, 2019), including activities such as gardening, nature walks and conservation activities. In July 2020 the UK Government invested 5.77 million in green social prescribing as a means of preventing and tackling poor mental health (NHS England, n.d.).

Whilst there has been significant investment in green social prescribing, evidence of the health and wellbeing benefits across the broad range of potential service users is limited. There is also a lack of evidence of the physiological health outcomes and the feasibility and acceptability of green social prescriptions are not fully understood. This Research Topic aimed to bring together articles focused on the feasibility of prescribing nature-based activities for the treatment of ill-health and to explore the range of health and wellbeing benefits that can be derived from participation in different green social prescriptions.

## Feasibility of prescribing nature-based social prescriptions

In line with the increased interest and demand for green social prescribing, Marx and More outlined the development of a formal referral pathway for nature-based interventions (the Green Health Prescription pathway) and explored the feasibility of the pathway through focus groups with healthcare professionals, service users, and nature-based intervention delivery partners. The focus groups highlighted that the barriers preventing referral to nature-based interventions included the sustainability of the pathway, funding, lack of awareness of existing nature-based interventions among practitioners, and the need to connect patients with appropriate interventions for their physical and mental health. These findings are in line with research by Wood et al. (2022) who highlighted a need for increased awareness of service provision amongst referring professionals. The strengths of the pathway included the easy-to-access “one-stop shop” for information, the convenience of a telephone hotline and links to other existing prescription pathways, as well the potential for peer support and empowerment.

The coronavirus pandemic adversely impacted the health of the population (Jaspal and Breakwell, 2020; Smith et al., 2020; Kwong et al., 2021; Wood et al., 2022) and resulted in disruptions to mental health services across the globe (Di Gessa et al., 2022). Fixsen and Barrett examined the challenges and opportunities of delivering green social prescribing during and in the aftermath of COVID-19 through interviews with social prescribers, general practitioners, managers, researchers and volunteers in Scotland and Northeast England. The findings revealed a diverse social prescribing landscape, with variation in schemes, funding, structure, and delivery. Whilst stakeholders agreed that there were numerous benefits to nature-based interventions; psychological and practical barriers, including health anxieties, mobility issues, and transport deficits were identified as barriers. It was also felt that work was needed to ensure older and multi-morbidity clients could fully engage with activities. These findings are also in line with those by Wood et al. (2022) who highlighted barriers to engagement with nature-based interventions such as transportation, mobility and health issues need to be considered to ensure more widespread access and use.

An increasing number of university students are also experiencing increasing poor mental health (Campbell et al., 2022). Boyd investigated the feasibility of using nature-based interventions with undergraduate students. Students took part in one of three interventions consisting of either a mobile app that directed users' attention toward nature, 2 weekly walks in nature, or a combination of the two. In focus groups, participants reflected on their experiences of the interventions and their attitudes toward nature on the university campus. The results revealed the need for a whole university approach, which considers the interventions and campus design simultaneously. The findings also highlighted the importance of green spaces for social purposes, as a shelter from the city environment, or for contact with wildlife. These findings support the use of wellbeing interventions together with green space development in universities.

## Health and wellbeing benefits of nature-based social prescriptions

Natural environments can act as a setting for a broad range of activities. Moula et al. undertook a systematic review of the impact of arts-based interventions delivered to children and young people in nature or outdoors spaces on nature connectedness, health, and wellbeing. Eleven studies were included in the review, with some focusing on creating art with materials found in nature and others using outdoor music making or forest school approaches. All studies revealed a positive impact of interventions on connectedness to nature and reported benefits for children's mental health and wellbeing, for example reduced stress and anger, increased energy, joy and emotional regulation. Four studies also reported increased engagement with learning processes, with outdoor environments supporting improved listening skills. Children with additional learning or behavioral needs, or with lower self-esteem also experienced additional improvements. However, the review also highlighted that quantitative research in the field was still in its infancy, supporting the need for larger experimental studies and upscaling of interventions.

Addressing the lack of physiological evidence of the benefits of nature-based interventions, Benz et al. investigated the impact of nature-based relaxation videos on heart rate and heart rate variability (HRV) using two sub-studies. In study 1, a 10-min nature-based relaxation video led to significant reductions in heart rate and increases in HRV in participants, indicating improved physiological health outcomes. In study 2, these improvements remained but were not significantly greater than improvements resulting from watching a meditation video or short clip from a popular movie. These findings highlight the need for further research into the physiological benefits of nature-based interventions and how these align with psychological health outcomes.

## Conclusions

This Research Topic provides further evidence of the feasibility and health benefits of nature-based interventions or green social prescribing activities. The studies included have demonstrated the need to overcome key barriers to prescription and engagement with nature-based interventions such as access and lack of awareness. There is also a need for larger experimental studies and upscaling of interventions to provide further evidence of health outcomes.

## Author contributions

CW and JR drafted the initial version of the editorial, which was then edited, revised, and agreed by all authors. All authors contributed to the editorial through acting as editors on articles submitted to the special edition and provided summaries of findings.
